# Identifying Barriers and Enablers to Attending Diabetic Retinopathy Screening in Immigrants to Canada From Ethnocultural Minority Groups: Protocol for a Qualitative Descriptive Study

**DOI:** 10.2196/15109

**Published:** 2020-02-12

**Authors:** Maman Joyce Dogba, Michael H Brent, Catherine Bach, Sarah Asad, Jeremy Grimshaw, Noah Ivers, France Légaré, Holly O Witteman, Janet Squires, Xiaoqin Wang, Olivera Sutakovic, Mary Zettl, Olivia Drescher, Zack van Allen, Nicola McCleary, Marie-Claude Tremblay, Stefanie Linklater, Justin Presseau

**Affiliations:** 1 Université Laval Québec, QC Canada; 2 University of Toronto Health Network Toronto, ON Canada; 3 Department of Family Medicine and Emergency Medicine Laval Unviersity Québec, QC Canada; 4 Ottawa Hospital Research Institute University of Ottawa Ottawa, ON Canada; 5 Women's Health College Toronto, ON Canada; 6 Ottawa Hospital Research Institute Ottawa, ON Canada

**Keywords:** retinopathy, diabetes, eye screening, theoretical domains framework, integrated knowledge translation, patient engagement, behavior change, immigrant health, minority health, patient oriented research

## Abstract

**Background:**

Immigrants to Canada belonging to ethnocultural minority groups are at increased risk of developing diabetes and complications, including diabetic retinopathy, and they are also less likely to be screened and treated. Improved attendance to retinopathy screening (eye tests) has the potential to reduce permanent complications, including blindness.

**Objective:**

This study aims to identify the barriers and enablers of attending diabetic retinopathy screening among ethnocultural minority immigrants living with diabetes in Quebec and Ontario, Canada, to inform the development of a behavior change intervention to improve diabetic retinopathy screening attendance.

**Methods:**

The research question draws on the needs of patients and clinicians. Using an integrated knowledge translation approach, the research team includes clinicians, researchers, and patient partners who will contribute throughout the study to developing and reviewing materials and procedures, helping to recruit participants, and disseminating findings. Using a convenience snowball strategy, we will recruit participants from three target groups: South Asian and Chinese people, and French-speaking people of African descent. To better facilitate reaching these groups and support participant recruitment, we will partner with community organizations and clinics serving our target populations in Ontario and Quebec. Data will be collected using semistructured interviews, using topic guides developed in English and translated into French, Mandarin, Hindi, and Urdu, and conducted in those languages. Data collection and analysis will be structured according to the Theoretical Domains Framework (TDF), which synthesizes predominant theories of behavior change into 14 domains covering key modifiable factors that may operate as barriers or enablers to attending eye screening. We will use directed content analysis to code barriers and enablers to TDF domains, then thematic analysis to define key themes within domains.

**Results:**

This study was approved for funding in December 2017, and the research ethics board approved the conduct of the study as of January 13, 2018. Data collection then began in April 2018. As of August 28, 2018, we have recruited 22 participants, and analysis is ongoing, with results expected to be published in 2020.

**Conclusions:**

Findings from this study will inform the codevelopment of theory-informed, culturally- and linguistically-tailored interventions to support patients in attending retinopathy screening.

**International Registered Report Identifier (IRRID):**

DERR1-10.2196/15109

## Introduction

Diabetes is among the most common chronic diseases worldwide. In Canada, the prevalence of diabetes is higher in immigrants to Canada (8.1%) than in those born in Canada (7.1%) [1] and varies across ethnic groups. Ethnicity and immigration can increase the risk of developing diabetes and related complications, with South Asians, Hispanic-Americans, Chinese, and Africans having a higher risk of developing diabetes and diabetes-related complications than Europeans [2,3].

For people living with diabetes worldwide, diabetic retinopathy is the most common complication and is also a critical microvascular complication [4-6]. This vascular disease of the retina comprises three forms: nonproliferative diabetic retinopathy, macular edema and ischemia, and proliferative diabetic retinopathy. Diabetic retinopathy is the leading cause of blindness among working-age populations in the Western world [2,5], and approximately one-third of people living with diabetes worldwide are already affected by retinopathy. In Canada, most people with type 1 and over half of those with type 2 diabetes will develop a degree of retinopathy in their lifetime [7].

Diabetic retinopathy screening can reduce the risk and progression of vision loss [8]. It is also one of the most effective and least costly ways to reduce the severe complications associated with this disease [9]. Screening (including an eye examination with pupil dilation using drops) by an optometrist, a general ophthalmologist, or a retina specialist helps to detect the disease earlier and thus increases the effectiveness of treatment [9]. Several international studies and guidelines recommend annual screening for diabetic retinopathy [2,10,11]. Guidance recommends that screening should be initiated five years after the onset of type 1 diabetes, and at the time of diagnosis with type 2 [12]. Despite these recommendations, however, diabetic retinopathy screening rates are much lower in ethnocultural minorities despite them having a higher prevalence of diabetic retinopathy [13]. Immigrants who are members of ethnocultural minorities are less likely to be screened and treated for diabetic retinopathy than nonimmigrant members of those same minority groups [14]. Obstacles to accessing eye care may differ between ethnic groups due to a variety of factors [15].

Low diabetic retinopathy screening rates are a public health issue [16] with considerable economic burden [8]. In Canada, diabetic retinopathy accounts for 25% of vision loss in people of visible minorities compared to 4% across all ethnicities [17]. Complications from diabetes account for 80% of the costs associated with the disease [18], which in 2015 was estimated to be Can$14 billion dollars (approximately US $10 billion) in Canada alone [19]. If left untreated, diabetic retinopathy leads to the continued use of multiple health care services [20]. The economic burden rests on the indirect costs of the progressive loss of sight due to decreased individual productivity, the use of counseling or rehabilitation services, and the government's income assistance policies in the event of total or partial incapacitation [21]. In addition to these broader economic and social costs, diabetic retinopathy is also associated with significant psychological and social consequences [8], including feelings of fear, depression, anger [22], shame, or guilt [23,24] related to the irregular management of diabetes. Improved attendance of diabetic retinopathy screening can reduce permanent complications of diabetes as well as the associated financial burden and negative psychosocial consequences [20].

We previously conducted a systematic review of barriers and enablers to attending diabetic retinopathy screening [25] and showed that such barriers and enablers had been investigated and described in a variety of ways. Framing diabetic retinopathy screening attendance as a health behavior could help researchers to understand the barriers and enablers to attendance for minorities in terms of the modifiable and nonmodifiable factors affecting this health behavior. Doing so may facilitate drawing upon theories of behavior and behavior change to provide a basis for developing a cumulative evidence base of factors that impact diabetic retinopathy screening attendance, and for developing interventions best suited to address these barriers and enablers.

Our review of barriers and enablers to attending retinopathy screening [25] identified 69 studies and highlighted recurring factors that may impede screening attendance:

- environmental context and resources factors (identified in 52 studies), including issues of access, competing priorities, economic concerns, schedule, referral problems, and specialist service availability;
- social influences (35 studies) related to doctor-patient communication, including language, trust, community support, and stigma;
- knowledge (35 studies) about diabetic retinopathy, of the difference between routine eye tests and screening;
- memory, attention, and decision processes (34 studies), including the lack of symptoms, competing comorbidities, and forgetting to attend;
- beliefs about consequences (26 studies) that screenings provide important health status information but worry about the harmful effects of screening;
- and emotions (23 studies), including fear, defensiveness, and adding to the overall demands of diabetes self-management, causing a feeling of being overwhelmed [25].

While these barriers may affect people living with diabetes in general, some barriers and enablers may be especially relevant to immigrants from ethnocultural minority groups. Furthermore, their experienced barriers/enablers may be underrepresented in the literature or not represented at all, leading to health care interventions that do not best serve their needs. Indeed, only 3/69 identified studies were conducted in Canada, and only one involved a minority group (First Nations Cree communities in Alberta). There is a need to better understand among the known barriers and enablers which are particularly salient among immigrants to Canada from different ethnocultural minority groups, and whether there are any as yet unidentified barriers that are relevant to specific groups. This will facilitate the development of services and support to meet their needs better and encourage higher screening attendance in those attending screenings at a lower rate.

We also completed a Cochrane systematic review of trials of interventions to improve retinopathy attendance [26]. Across the 66 trials identified (only 3 in Canada) [26], 56 of which compared intervention to usual care, screening attendance increased by 12% (risk difference 0.20 [95% CI 0.10-0.14]) [26]. We showed that specific behavior change techniques were associated with improved screening attendance, including goal setting (outcome) (0.26 [95% CI 0.16-0.36]) and feedback on outcomes of behavior (0.22 [95% CI 0.15-0.29]) in interventions targeting patients [26]. The review guides potential high-yield strategies for future implementation trials in principle, but these likely need to be tailored to address relevant barriers in different subgroups to ensure optimal fit between barriers and the interventions designed to address them [27]. Rather than assuming a generic intervention will solve this problem, a collaborative and patient-oriented approach is needed to understand how different vulnerable populations with a higher risk of diabetic retinopathy experience barriers to attending the screening.

A comprehensive theory-based approach may be particularly useful in providing a breadth and depth of factors to explore as barriers and enablers to screening attendance, which, for our study, is the Theoretical Domains Framework (TDF) [28,29]. The TDF synthesizes prevailing theories of behavior and behavior change, and the constructs within them, into a set of 14 theoretical domains that represent a breadth of factors demonstrated to affect or impact behavior and behavior change. The domains include knowledge, skills, social/professional role and identity, beliefs about capabilities, optimism, beliefs about consequences, reinforcement, intentions, goals, memory/attention/decision processes, environmental context and resources, social influences, behavioral regulation, and emotion. It has been previously used to understand the barriers and enablers to behavior across a range of settings and populations, including patients [30-33], was included in our systematic review of barriers and enablers to attending diabetic retinopathy screening [25], and will serve as the basis for identifying barriers and enablers in this study.

We aim to identify barriers and enablers to attending diabetic retinopathy screening in ethnocultural minorities, such as immigrants from South Asia or China and Francophone immigrants of African descent [34], in the provinces of Ontario and Quebec, Canada.

## Methods

### Study Approach

This study will use an integrated knowledge translation [35] approach, which involves partnering with clinicians and people living with diabetes at all stages of research. This includes the steps of defining or refining research objectives, collecting and analyzing data, disseminating results, and developing policies. An integrated knowledge translation approach better ensures that research incorporates the experiences and needs of patients. In this study, we plan to have up to 12 patient partners included in the research process. At least six people (two from each selected subgroup of immigrants from South Asia or China, or Francophone immigrants of African descent) will be members of the research team. They will be invited to take part in the discussions and decision-making processes as key members of the research team, contributing their expertise through their experience living with diabetes and accessing and attending diabetic retinopathy eye screening. The patient partners will be as diverse in age and gender as possible to help ensure a broader scope of opinions and experiences.

### Theoretical Framework

The TDF will inform the content of the interview topic guides and subsequent directed content analysis. This will allow us to identify key theoretical domains likely to influence diabetic retinopathy screening attendance within each of our three subgroups. The use of the TDF will provide the capacity to draw upon evidence from the broader literature on barriers and enablers to diabetic retinopathy screening attendance, as well as the wider behavioral science literature. The use of a framework thus ensures contribution to a cumulative evidence base on modifiable factors to address to encourage higher attendance of retinopathy screenings.

### Design

We defined the health behavior under study using the Target-Action-Context-Time principle: immigrants to Canada from South Asia or China, or of African descent with any diabetes (target) attending (action) diabetic retinopathy screening with an eye specialist (context) in the next year (time). We will then use the TDF to identify barriers and enablers to attending retinopathy in each ethnocultural group. We will conduct a descriptive qualitative study guided by the TDF. The qualitative approach provides a global perspective and interpretative understanding of the phenomenon while allowing theoretical and methodological adjustments throughout the research process. This ensures that we capture the breadth of potentially modifiable factors to inform subgroup-specific intervention development. We will use consolidated criteria for reporting qualitative research reporting guidelines [36] to report our eventual findings from this study.

Consistent with guidance [37], the TDF will be used as a basis for describing the topics to be addressed in the interview guide and to inform the analysis. The guide will be developed, and findings interpreted with our patient partners to ensure consistency with any ethnocultural features of interest. While the TDF will provide the theoretical structure, we will incorporate open questions within the interview guides and an inductive theme-generation process for factors that may not directly fit within the TDF. See Multimedia Appendix 1 for examples of our topic guide.

### Context

This study will take place in the provinces of Ontario and Quebec in Canada and will focus primarily on recruiting in Toronto, Ottawa, Montreal, and Quebec City. We may add other areas depending on referrals. Local team members will facilitate the selection of locations for data collection and recruitment of participants in collaboration with primary care (eg, family practice teams) and community organizations in both provinces. We will also collaborate with Diabetes Action Canada (DAC) and its Diabetic Retinopathy, Patient Engagement, and Knowledge Translation groups. DAC is one of five pan-Canadian research networks focused on a chronic illness and seeks to improve the lives of people living with diabetes through patient engagement and collaboration.

### Participants

Study participants will be immigrants to Canada from South Asia or China, and French-speaking immigrants of African descent living with diabetes. While Canada is home to immigrants from many nations, these three ethnocultural groups represent the majority of ethnocultural minorities currently arriving in Canada annually [38] who have a higher prevalence of diabetic retinopathy. These groups also align with our study team members’ linguistic abilities to ensure that interviews can be conducted with participants in their native language. The Francophone immigrants of African descent have been selected as they represent more than three-quarters of the Francophone immigrants that arrived in Canada in 2014 [39]. The term immigrants refers to “individuals who moved from their country of origin into a new country for the purpose of resettlement” [40]. This definition, according to the International Organization of Migration, includes those who arrive and stay through an irregular migration process such as “temporary foreign workers, foreign students, refugees and other involuntary migrants” [40].

This study will not include refugee populations since they experience unique barriers in accessing health care and health coverage. In our analysis, we will distinguish between recent immigrants (living in Canada for less than five years) and immigrants who have been established in the country for a longer period. Inclusion criteria for participation in the study include: (1) at least 18 years of age; (2) an immigrant from South Asia (native speakers of Urdu or Hindi) or China (native speakers of Mandarin), or a Francophone immigrant of African descent; (3) living personally with diabetes; and (4) agree to participate in the study. The exclusion criterion is to have a confounding severe ocular disease other than diabetic retinopathy, such as cataracts or glaucoma.

### Participant Recruitment

Study participants will be recruited through the patient circles of Diabetes Action Canada, our team members’ networks, primary care units, and community organizations serving immigrants or people living with diabetes in Ottawa, Toronto, Quebec City, and Montreal. We will contact selected organizations by phone or email to introduce ourselves and the research project.

We will establish contact with the primary care units and community organizations via a specified contact person working at each establishment. We will ask community organizations interested in collaborating with us to support the recruitment process by sharing recruitment messages on their websites, in their newsletters, and via email to clients of their organization. We will also ask to post a recruitment announcement on their premises and assist us in accessing a private space to conduct interviews with participants. This is a documented method for working with vulnerable and underserved groups and likely also to promote reaching groups who are medically underserved as well [41].

To date, we have partnered with Polycultural [42] in Toronto and Alliance des communautés culturelles pour l’égalité dans la santé et les services sociaux (ACCÉSSS) in Montreal for this study. They will assist us in recruiting participants and provide private workspace(s) to conduct individual interviews with participants if needed. We will continue to involve further community organizations as needed. We will also approach family doctor leads in primary care units regarding their interest in informing relevant patients about our research project and making study information, including recruitment text, available at their location(s). People who are interested in learning more about our study or who wish to participate will be invited to contact the study coordinators. People meeting all inclusion criteria will be selected to participate. If the number of people willing exceeds our needs, we will select participants using the maximum variation sampling procedure. We will provide Can$40 (US $30.40) as financial compensation in recognition of their participation and reimburse participants for travel costs.

### Sampling

We will use a purposive sampling approach supplemented with snowball sampling. We will aim to recruit 13-20 people per subgroup (South Asian immigrants, Chinese immigrants, and Francophone immigrants of African descent). Purposive sampling will involve aiming to recruit participants according to a sampling frame that aims to balance recruitment across sex/gender, age, time since immigration (within the last five years or more than five years), and reported diabetic retinopathy screening attendance within the last year or not. This will facilitate comparing results between recent immigrants and those who have been in Canada for a longer time, as well as between people who attend/do not attend regular diabetic retinopathy screening.

Interviews will continue in each subgroup until thematic data saturation is reached, using the “10+3 rule” [19] whereby at least ten interviews in each subgroup will be conducted, followed by a further three interviews, and if the last three do not bring up new barriers/enablers, saturation will be judged to have been achieved. If a new barrier/enabler is identified, interviews will continue until three consecutive interviews do not bring up anything new. Based on previous studies, we expect to achieve saturation in each subgroup within 13-20 interviews [43].

### Interview Procedure and Data Collection

A semistructured interview guide has been developed, based on the TDF, to identify barriers and enablers to attending diabetic retinopathy screening. One-on-one interviews (or accompanied by family/friend/caregiver, if appropriate) will be conducted by native speakers of each language (Hindi, Urdu, French, and Mandarin). Interviews will be conducted over the phone or, when feasible, in person, and will be approximately one hour in duration. The guide will be developed per published standards [37]. Interview topic guides will be initially developed in English, then translated into the languages mentioned above. Transcribed interviews will be reviewed by team members (research staff and patient partners) who speak the respective languages to ensure both retention of the theoretical meaning for each domain and, where appropriate, ethnocultural adaptations are maintained. In keeping with our integrated knowledge translation approach, we will include patient partners’ input in developing and refining the interview guide.

### Analysis Plan

Individual interviews will be audio-recorded, translated into English, transcribed, then analyzed using NVivo (QSR international, Doncaster, Australia). Transcripts will be analyzed using directed content analysis [44], a deductive approach that uses an established theoretical framework as a basis for coding responses. We will develop a coding manual and then independently double code barriers and enablers expressed by respondents into the TDF domain(s) best reflecting their views while leaving open the possibility that barriers/enablers might not fit into a given domain. Once views have been coded to TDF domains, we will conduct an inductive thematic analysis within each domain to identify specific emergent themes. The result will provide an indicator of key domains to target within each group for the development of a future intervention to support attendance to retinopathy screening. Consistent with established criteria and guidance for using the TDF, key domains will be determined based on how strongly expressed the barriers/enablers are by respondents, how often a given domain is represented, and whether there are discrepancy views between participants [32]. Any barriers and enablers not fitting within TDF domains will be identified and thematic analysis conducted.

## Results

This study was approved for funding in December 2017, and the research ethics board approved the conduct of the study as of January 13, 2018. Data collection then began in April 2018. As of August 28, 2018, we have recruited 22 participants, and analysis is ongoing, with results expected to be published in 2020.

## Discussion

This study will be among the first to explore barriers and enablers to attending retinopathy screening in ethnocultural minority immigrants living with diabetes from different socio-cultural backgrounds. It will allow us to determine whether and how barriers/enablers vary by group and whether they differ from known barriers/enablers elicited from the general population of people with diabetes. Should more specific barriers be identified, this would argue in favor of developing interventions tailored according to socio-cultural background. However, should similar barriers emerge as those of the wider population, this would suggest more general intervention strategies are needed.

Results from this study will also help to develop behavior change intervention strategies that are ready for rigorous evaluation. In a future study based on this project, our patient partners and we hope to work alongside Diabetes Action Canada’s Patient Engagement, Diabetic Retinopathy, and Knowledge Translation groups to codevelop intervention components that address barriers identified in this study. We will consider feasible options for how to deliver the intervention (methods of delivery), by who and to whom, where, and how often, informed wherever possible by existing literature on effective strategies for particular barriers. Once prototype intervention materials and approaches are developed, we will evaluate reactions and feedback to intervention materials and processes [45].

Many people from minority ethnocultural groups who have immigrated to Canada are at higher risk of the complications of diabetic retinopathy, but to date, we know very little about the modifiable factors that impact their screening attendance. The present study aims to fill this gap across multiple groups, and through an active process of involvement and engagement, ensure that the research is conducted in a manner that best reflects the experiences of members of each of the three groups. We hope that this approach may also serve as an exemplar to other planned research involving similar under-represented groups to ensure that the foundations for developing interventions to improve care consider the perspectives of these groups.

## Appendix

Multimedia Appendix 1 Woman from ethnocultural minority undergoing a diabetic retinopathy screening.

## Abbreviations

ACCÉSSS: Alliance des communautés culturelles pour l’égalité dans la santé et les services sociaux
DAC: Diabetes Action Canada
TDF: Theoretical Domains Framework
