# Supplementary material for: Identifying Barriers and Enablers to Attending Diabetic Retinopathy Screening in Immigrants to Canada From Ethnocultural Minority Groups: Protocol for a Qualitative Descriptive Study
Source: JMIR Res Protoc. 2020 Feb 12;9(2):e15109. doi: 10.2196/15109 (PMC7055809; doi:10.2196/15109)
Supplement: Multimedia Appendix 1 [file resprot_v9i2e15109_app1.pdf]

INTERVIEW GRID

Identifying barriers and enablers to attendance to diabetic retinopathy screening

Thanks for speaking with me today. I'm (name) and I'm part of a research team that is interested in the views of people with diabetes about going to get their eyes screened every year. We are trying to make sure the healthcare system is best designed to ensure people get the care and support they need, and we need your views and opinions – any comments will help! Most of my discussions with other people last 45-60 minutes.

If you agree, I will audio record our discussion to make sure I capture all your thoughts. Don't worry; all identifying information mentioned (your name/names of other people or places) will be removed from the final transcript so no one will be able to link what you say back to you specifically. If you want to take a break or stop or if you wish to withdraw from the study you are completely free to do so at any point. All good so far?

So as I mentioned, I am interested in your thoughts about a specific type of eye screening called "diabetic retinopathy screening", and your thoughts about what stops and helps you to attend this screening. Don't worry if you don't know what diabetic retinopathy or its screening are, I will explain these to you in a minute. But before I do, I just want to say that there really are no right or wrong answers to the questions; I'm really just interested in your honest opinions and thoughts. Again your views will stay completely confidential. I am not a doctor, I work with the research team and today I am here strictly to gather your valued input on this topic through a series of questions to help us discuss your views.
Do you have any questions before we start?

Give the consent form to the participant.
When signed, start the voice recorder.

State: Date of the interview. Name of the interviewer and of the participant.

1. Some people find that they can manage their diabetes more or less challenging. I'm interested to know how you feel about your experience so far living with diabetes. How comfortable are you in managing your diabetes? Why? [DOMAIN: EMOTION]

PROMPTS:

- What are some steps you take in managing your diabetes? For example, in terms of nutrition; physical activity; weight management; control of blood glucose, blood pressure and cholesterol; regular screening for nerve issues (ex. numbness or weakness in hands or feet) and kidney problems?
- How do you find integrating the foods you eat from [say their country] with managing your diabetes? Any issues?

2. Have you ever attended an education session for managing your diabetes? [DOMAIN: Skills]

PROMPTS:

- If so: did they cover eye screening?
- If so: when was the last time you attended?

3. Now a bit more specifically, what do you know about diabetes and your eyes? Diabetic retinopathy? Diabetic retinopathy eye screening exam? [DOMAIN: Knowledge]

PROMPTS:

- Have you heard that diabetes can affect your eyes? What have you heard?
- Have you heard about diabetic retinopathy? What is it?
- How did you come to learn about it? Have you heard about the possible complications associated with diabetic retinopathy?
- What do you think is the purpose of this type of eye screening?
- Who do you think should have their eyes screened and why?

- Where do you go for retinopathy screening?

To be sure we are referring to the same thing regarding the diabetic retinopathy and the eye screening exam, I will briefly describe what it is.

Diabetic retinopathy is a complication that people with diabetes could get, and it affects the eyes. Diabetes can cause changes in blood vessels, including the small blood vessels in the back of the eye – retina – that can lead to partial loss of vision and blindness in the most severe cases. Early and frequent eye screening is the key to management.

Retinopathy eye screening exam is part of an eye examination performed by eye doctors including optometrists, general ophthalmologist or a retina specialist. It is a different eye exam than for prescription glasses, though it might be done during the same appointment. What is unique about retinopathy screening is that it involves what is called a "dilated eye exam", where an eye specialist applies drops in a patient's eyes to dilate his or her pupil (making the pupil/the black part of the eye very large). The patient has to wait about 30 minutes for the drops to work and vision will get blurry, and then the eyes stay that way for a few hours so often need to avoid direct sunlight after the appointment.

Once the pupils are dilated, a microscope such as this one *(show the picture of the Slit-Lamp in the appendix on page 10 through 12*) is the most common equipment used in retinopathy eye screening to examine the structure inside the patient's eye. With special equipment a set of pictures is also sometimes taken.

- *Appendix 1 (p.10): Participants from South Asia*
- *Appendix 2 (p. 11): Participants from China*
- *Appendix 3 (p. 12): Participants of African descent*

4. To the best of your knowledge, have you had a diabetic retinopathy screening exam in the last year? [DOMAIN: Nature of behaviour]

PROMPTS:

- If not, why? What are the reasons?
- If so, where? Who performed the screening exam?
- How many times have you been screened in the last 2 years?

5. Did a health specialist recommend you to be screened for diabetic retinopathy? [DOMAIN: SOCIAL INFLUENCES]

PROMPT:

- If so, did you follow their recommendation? [DOMAIN: Nature of the behaviour]

6. If you already get screened, can you walk me through the process and steps involved in you getting your eyes screened? [DOMAIN: KNOWLEDGE – Procedural knowledge]

PROMPTS:

- Were you referred by someone? If so, who did?
- Who made your appointment?
- Where was the eye screening appointment?
- How did you get there?
- Overall, what are some of the reasons that helped you to make sure you got your eyes screened in the last year?
- If you wanted to get your eyes screened or if you thought it was time to get your eyes screened, what would you do?
- Anything else?

For the rest of the discussion, please thinking about "you personally going to see an eye specialist to have your eyes screened for diabetic retinopathy within the next year" when responding to my questions.

7. What are some of the benefits (good things) that you see in attending diabetic retinopathy screening within the next year? [DOMAIN: Beliefs about the consequences]

PROMPTS:

- For you personally? For someone else (e.g., someone in your family)?

8. What are some of the bad things (concerns, issues, inconveniences) that might happen when/if you attend diabetic retinopathy screening within the next year? [DOMAIN: Beliefs about the consequences]

PROMPTS:

- For you personally? For someone else (e.g. someone in your family)?
- Do the positives outweigh the negatives in attending?

9. How strongly do you want to attend diabetic retinopathy screening within the next year? Why? [DOMAIN: Intention]

PROMPT:

- Could you tell me a bit about why you want (or don't want) to?
- Are you inclined to consult a doctor or any physician when you may have a health problem to treat? Would you say it comes from a personal preference or a cultural habit? [DOMAIN: Nature of behaviour]

10. How confident are you that you will be able to attend eye screening for diabetic retinopathy within the next year? [DOMAIN: Beliefs about capabilities]

PROMPTS:

- What issues reduce your confidence that you will attend? (e.g. Difficulty of getting referred? Transportation? Financial issue? Child care issue? Taking time off work/ school? Others?)

- Any language-related factors (for yourself or the healthcare providers) make you feel more or less confident that you will attend?
    - How do you feel about your ability to *ask questions* when attending an eye screening appointment for diabetic retinopathy? [DOMAIN: Skills – interpersonal]
    - How do you feel about your ability *to respond to your healthcare providers' questions* when attending an eye screening appointment for diabetic retinopathy? [DOMAIN: Skills – interpersonal]
- What makes you feel more confident that you will be able to attend?

11. How does attending diabetic retinopathy screening within the next year fit with your schedule/agenda? [DOMAIN: Goals]

PROMPTS:

- How much of a priority is it to you? What are some higher priority things you need to do? Do any of those potentially get in the way of attending diabetic retinopathy screening?
- Does anything else get in the way? If so, what?

12. Do you expect that attending screening for diabetic retinopathy will result in more good things than bad things for yourself? [DOMAIN: Optimism]

13. If you take a moment to imagine yourself at your next eye screening, right before doing the test, what emotions come to mind? [DOMAIN: Emotions]

PROMPTS:

- How do these feelings influence whether you attend a diabetic retinopathy screening?
- What positive emotion(s) do you associate with attending a diabetic retinopathy screening?
- What negative emotion(s)?

14. What are the reasons people with a similar cultural background as you may not attend their eye screenings?

(Follow up: is there anything about being from an [x] background yourself that influences whether you attend your eye screening?) DOMAIN: Social/professional role and identity]

PROMPTS:

- Does attending an eye screening exam challenge any of your values? If so, how?
- Some people may have other issues (e.g. ethical or cultural beliefs) with regards to attending this screening or not. Is this something that personally influences whether you attend screening for diabetic retinopathy?
- Some people have religious or spiritual beliefs that influence whether they go or do not go to retinopathy screening. Is this something that influences whether you attend screening for diabetic retinopathy?
- From your perspective, whose responsibility is it to make sure that you go to your retinopathy screening within the next year? (Self? Family? Friend? Doctor?)

15. Can you tell me a bit about whose views are important to you when attending diabetic retinopathy screening within the next year? [DOMAIN: Social influences – Subjective norms]

PROMPTS:

- Family, friends, co-workers, physician, people around you, etc.
- Are there people in your life who motivate or discourage you to be screened?
- Who would influence your decision the most?

16. Do you have any family, friends, colleagues who have been diagnosed with diabetes and have been screened for diabetic retinopathy? [DOMAIN: Social Influences - Descriptive norms]

PROMPTS:

- If so, have you talked with them about their experiences?
- How did their experience influence whether you would attend the screening?

17. What resources do you use and need to make sure you can attend screening for diabetic retinopathy within the next year? [DOMAIN: Environmental context and resources]

PROMPTS:

- Any logistical issues to you attending? Are you comfortable calling the eye specialist office? [DOMAIN: Beliefs about capabilities]
- Any issues in you getting referred to attend?
- Waiting times before the date of your appointment? In the waiting room the day of your appointment?
- Is the location of the screening an issue?
- Any financial issues? (Do you have insurance to cover it?)
- Any transportation issues?
- Any childcare/time off work issues?
- Any issues about having enough time?

18. Are there any rewarding experiences in the past that make you want to go get your eyes screened for retinopathy within the next year? [Reinforcement]

PROMPTS:

- Are there any negative experiences in the past that make you not want to go have your eyes screened within the next year? What about if you do not attend the screening?

19. Have you ever forgotten to attend an appointment for eye screening? Or any medical appointment? What might help you to remember to go within the next year? [DOMAIN: Memory, Attention, Decision processes]

PROMPTS:

- Like most people you probably have lots going on in your everyday life. Can you talk to me about how you “block” out distractions and competing demands to attend screening?
- Do you ever have trouble deciding whether to get your eyes screened or not?

20. Is there anything specific that is already in place, or strategies that you already use, that will help you to make sure you will go to your screening within the next year? What strategies would help other people living with diabetes? [DOMAIN: Behavioural Regulation]

To close:

- ❖ Thinking about everything that we talked about, what are the most important things that determine whether you will go to get your eyes screened for retinopathy within the next year?
- ❖ What could be done to make it easier for you to get your eyes checked?
- ❖ Is there a particular reason why you agreed to participate in this study?
- ❖ Any final thoughts?

Thank you very much for your time and participation.

APPENDIX 1

Pictures of the Slit-Lamp

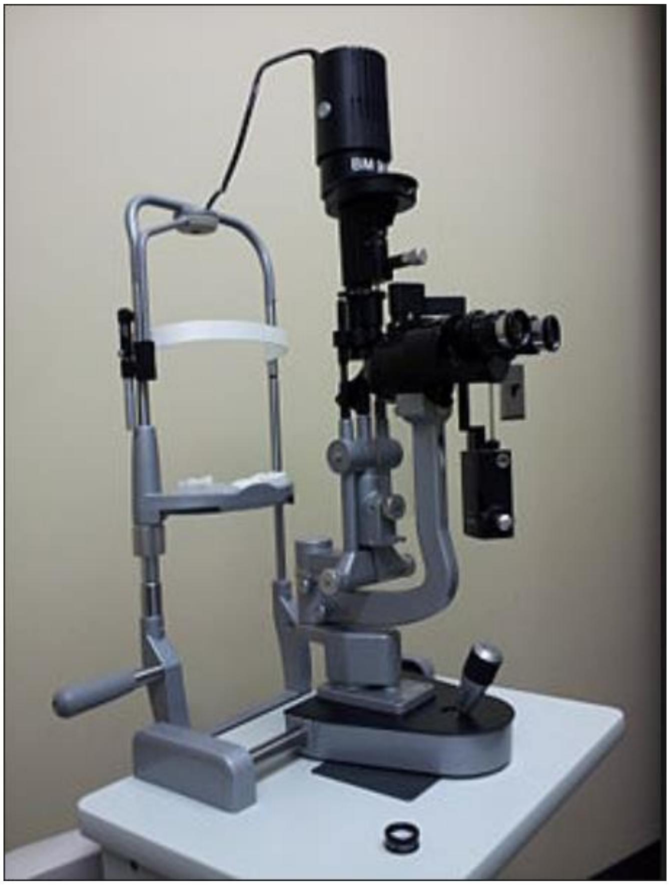

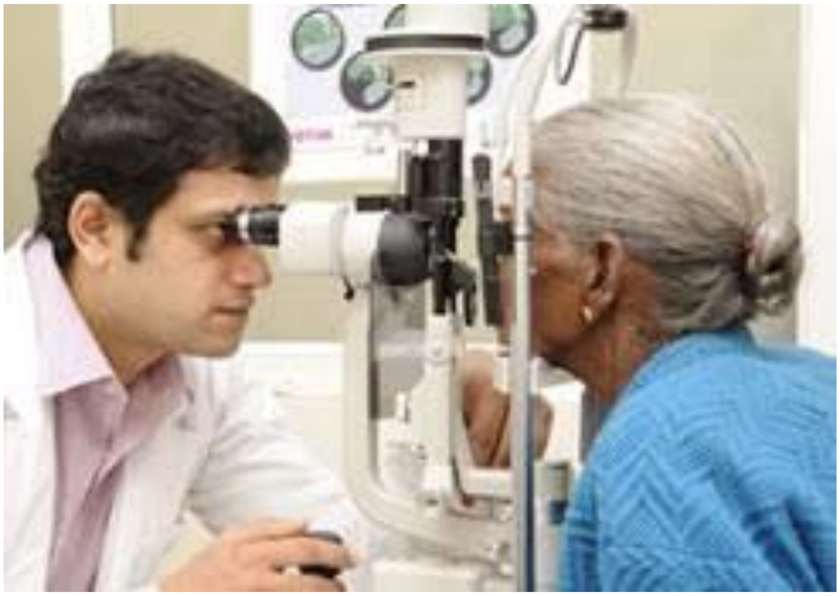

APPENDIX 2

Pictures of the Slit-Lamp

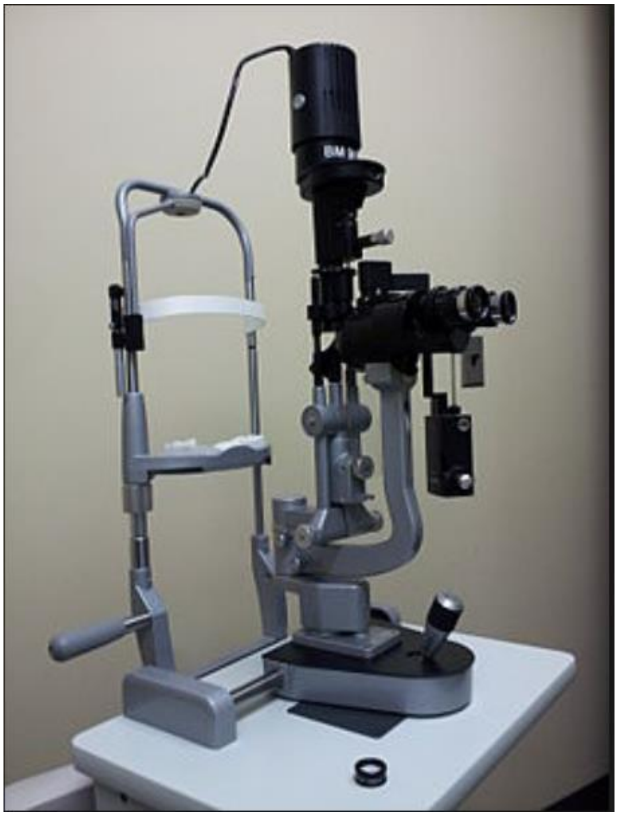

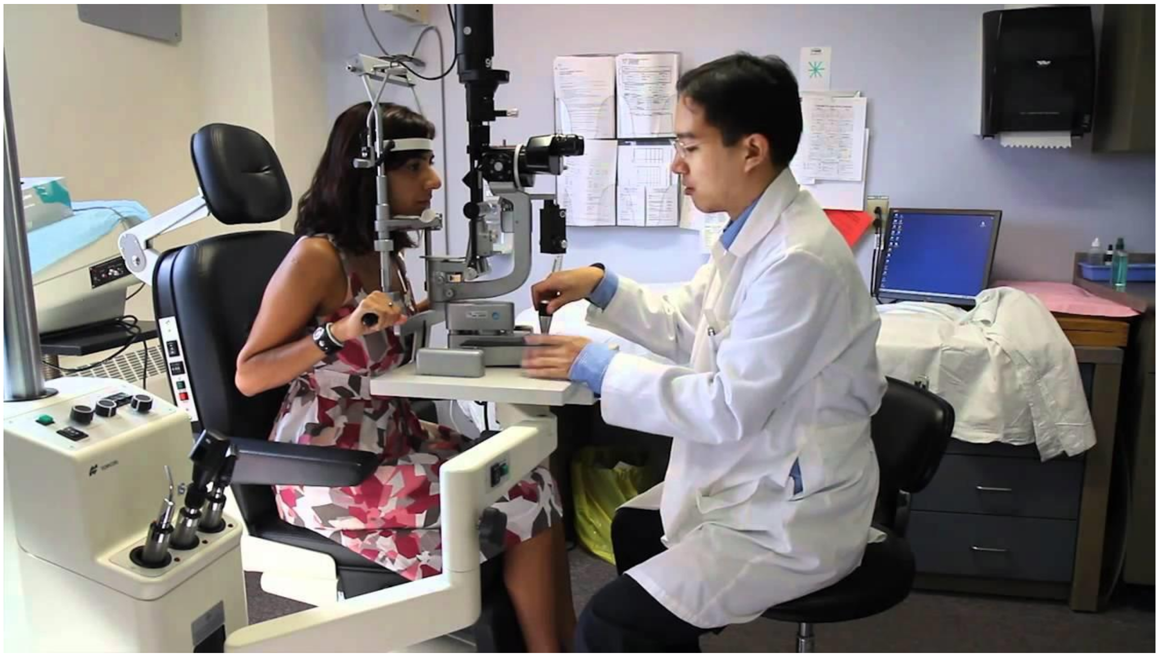

APPENDIX 3

Pictures of the Slit-Lamp

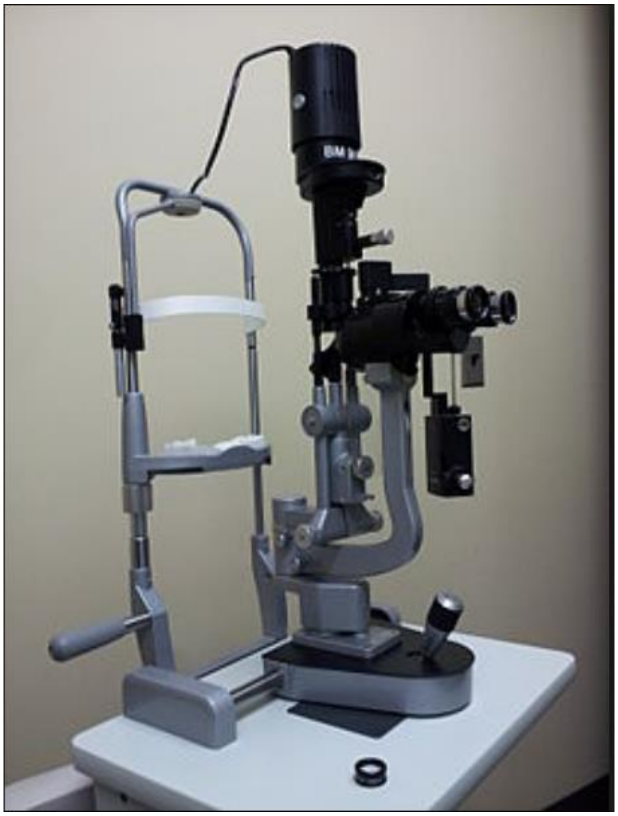

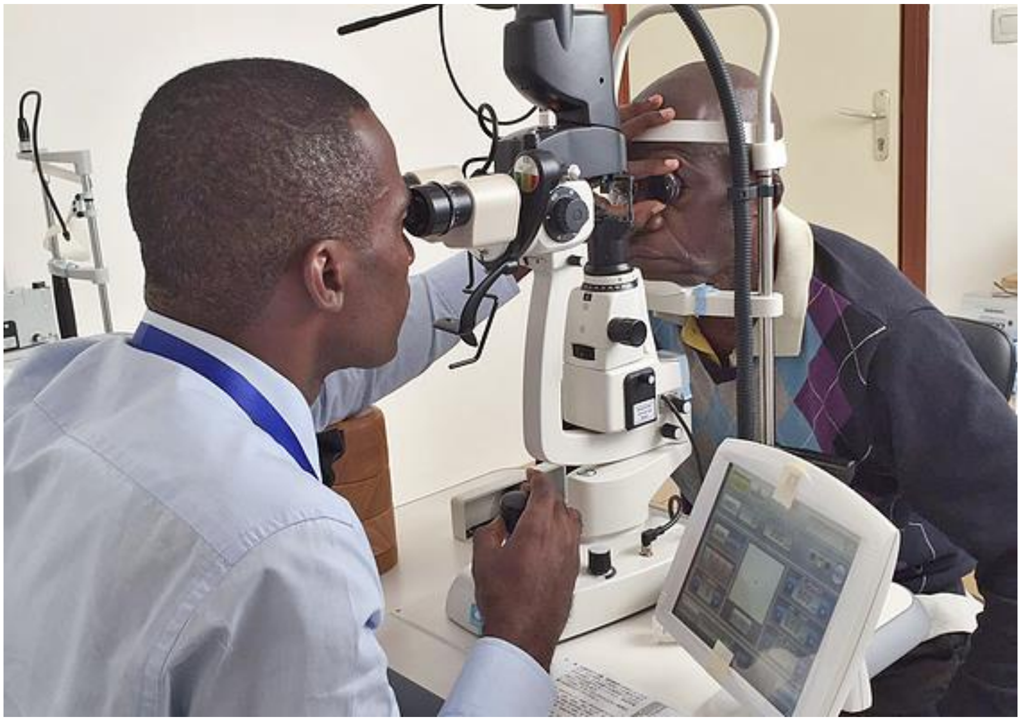

PERSONAL INFORMATION

Providing us with the following information will help us to describe who took part in our discussions at an overall level. Your responses will be combined with everyone else's so that you will not be identifiable. Thank you!

1) Sex that you were assigned at birth:

☐ Male
☐ Female

2) What is your year of birth? ____________________

3) Which type of diabetes do you have?

☐ Prediabetes
☐ Type 1
☐ Type 2
☐ Gestational diabetes
☐ Other, please specify: _____________________

4) Approximately what year did you receive your diabetes diagnosis?

____

5) In which country did you receive your diagnosis? ____________________

6) In what year did you arrive in Canada? ____________________

7) What best describes your ethnic origin? _________________________

8) What is your country of origin (the country in which you were born)?

____

9) What is your marital status?

☐ Single
☐ Married
☐ Common-law
☐ Separated/Divorced
☐ Widowed
☐ Prefer not to say

10) What is the size of your household?

☐ I live on my own

☐ I live with _________ roommate(s)

☐ I live with _________ family members (including myself and including children)

11) What is your employment status?

☐ Full time job

☐ Part time job

☐ Unemployed and seeking employment

☐ Unemployed and not seeking employment

☐ Retired

☐ Other: ____________________

☐ Prefer not to say

12) What is the approximate total annual income of your household (the sum of the annual income of all family members/people living with you, before taxes) in CND dollars?

☐ Less than $19 999

☐ $20 000 to $39 999

☐ $40 000 to $59 999

☐ $60 000 to $79 999

☐ $80 000 or more

☐ I prefer not to answer

☐ I don’t know

13) What is your highest level of education?

☐ Elementary school

☐ High school

☐ Trade school

☐ College

☐ Undergraduate school (Baccalaureate)

☐ Graduate school

☐ Other: ____________________
